# Seroprevalence and molecular characteristics of hepatitis E virus in household-raised pig population in the Philippines

**DOI:** 10.1186/s12917-015-0322-z

**Published:** 2015-01-27

**Authors:** Xiaofang Liu, Mariko Saito, Yusuke Sayama, Ellie Suzuki, Fedelino F Malbas, Hazel O Galang, Yuki Furuse, Mayuko Saito, Tiancheng Li, Akira Suzuki, Hitoshi Oshitani

**Affiliations:** Department of Virology, Tohoku University Graduate School of Medicine, 2-1 Seiryo-machi, Aoba-ku, Sendai, Miyagi 980-8575 Japan; Tohoku-RITM Collaborating Research Center on Emerging and Reemerging Infectious Diseases, RITM compound, FCC, Alabang, Muntinlupa City, 1781 Philippines; Research Institute for Tropical Medicine, FCC, Alabang, Muntinlupa City, 1781 Philippines; Depatment of Virology II, National Institute of Infectious Diseases, 4-7-1Gakuen, Musashimurayama-shi, Tokyo 208-0011 Japan

**Keywords:** Hepatitis E virus, Household-raised pig, Seroprevalence, Genotype 3, Philippines

## Abstract

**Background:**

Hepatitis E virus (HEV) infection is a significant public health concern in Asia, and swine is an important source of sporadic HEV infection in human. However, no epidemiological data are available regarding HEV infection among the swine or human population in the Philippines. To assess the HEV infection status among pigs in rural areas, we investigated the molecular characteristics and seroprevalence of HEV among household-raised pigs in San Jose, Tarlac Province, the Philippines.

**Result:**

Serum and rectal swab samples were collected from 299 pigs aged 2–24 months from 155 households in four barangays (villages) between July 2010 and June 2011. Enzyme-linked immunosorbent assay (ELISA) revealed that 50.3% [95% confidence interval (CI) 44.5–56.2%] and 22.9% (95% CI 18.2–28.1%) of pigs tested positive for anti-HEV IgG and IgM, respectively. HEV RNA was detected in the feces of 22 pigs (7.4%, 95% CI 4.7–10.9%). A total of 103 households (66.5%, 95% CI 58.4–73.8%) had at least one pig that tested positive for anti-HEV IgG or IgM or HEV RNA. The prevalence of anti-HEV IgG and IgM in breeding pig (8–24 months) were higher than that in growing pigs (2–4 months) (p < 0.0001 and p = 0.008, respectively). HEV RNA was more frequently detected in 2–4-month-old pigs (9.2%, 95% CI 5.4–14.6%) than in ≥5-month-old pigs (4.8%, 95% CI 1.1–8.5%) without statistical significance (p = 0.142). HEV RNA showed 0–27.6% nucleotide difference at the partial ORF2 gene among the detected viruses, and a majority of them belonged to subtype 3a (20/22, 90.9%).

**Conclusion:**

We found a high prevalence of HEV antibodies in the household-raised pig population in rural areas of the Philippines, which indicates the potential risk of HEV infection among local residents. Only genotype 3 of HEV was observed, and genetically diverse strains of HEV were found to be circulating in pigs in this study.

**Electronic supplementary material:**

The online version of this article (doi:10.1186/s12917-015-0322-z) contains supplementary material, which is available to authorized users.

## Background

Hepatitis E was first documented as a unique clinical entity distinct from hepatitis A and B in water-borne epidemic hepatitis in India in 1978 [1]. Hepatitis E virus (HEV), the sole member of genus *Hepevirus* in the *Hepeviridae* family, is the causative agent of self-limited or fulminant hepatitis [2]. The virion of HEV is spherical, nonenveloped, 27–34 nm in diameter, with a single-stranded, positive sense RNA genome. The RNA is approximately 7300 nucleotides in length and contains three open reading frames (ORFs). ORF1 encodes nonstructural proteins, while ORF2 encodes capsid proteins and ORF3 encodes a small protein of unknown function [3]. Mammalian HEV falls into four major genetically distinct genotypes based on nucleotide differences [4-6]. Genotypes 1 and 2 are the most common causes of epidemic hepatitis in humans in tropical and subtropical countries with poor sanitation and unsafe water supply [1,7]. Genotypes 3 and 4 are considered to be of zoonotic origin and are together recognized as an important cause of sporadic hepatitis cases in humans both in developing and industrialized countries [6,8,9].

Some evidence indicates that pigs are an important source of zoonotic HEV genotypes 3 and 4. Case reports have shown that viruses recovered from clinical patients with hepatitis E and the consumed pork were genetically similar [8,10]. A cluster of human isolates from autochthonous hepatitis E cases were found to be genetically similar to the local swine strains by phylogenetic analysis [11]. Meta-analysis of 10 cross-sectional studies revealed greater chances of HEV seropositivity in people with occupational exposure to pigs than in the general human population [12].

HEV genotype 3, which was first isolated in 1997 [6] from domestic pigs in the United States, has been shown to be widely distributed in pigs in all continents. Genotype 4 was first reported in China [5,9], and it appears to be present in pigs and humans exclusively in Southeast Asia. Recently, however, genotype 4 has been detected in pigs and in human cases with more severe clinical manifestations than those with other HEV genotypes in Europe [13,14]. Genotypes 3 and 4 are quite diverse and can be further classified into 10 (3a–3j) and seven (4a–4 g) subtypes, respectively, on the basis of five different regions of HEV, including 5994–6294 nucleotide positions of ORF 2 (GenBank accession number M73218) [4].

**Figure 1:**
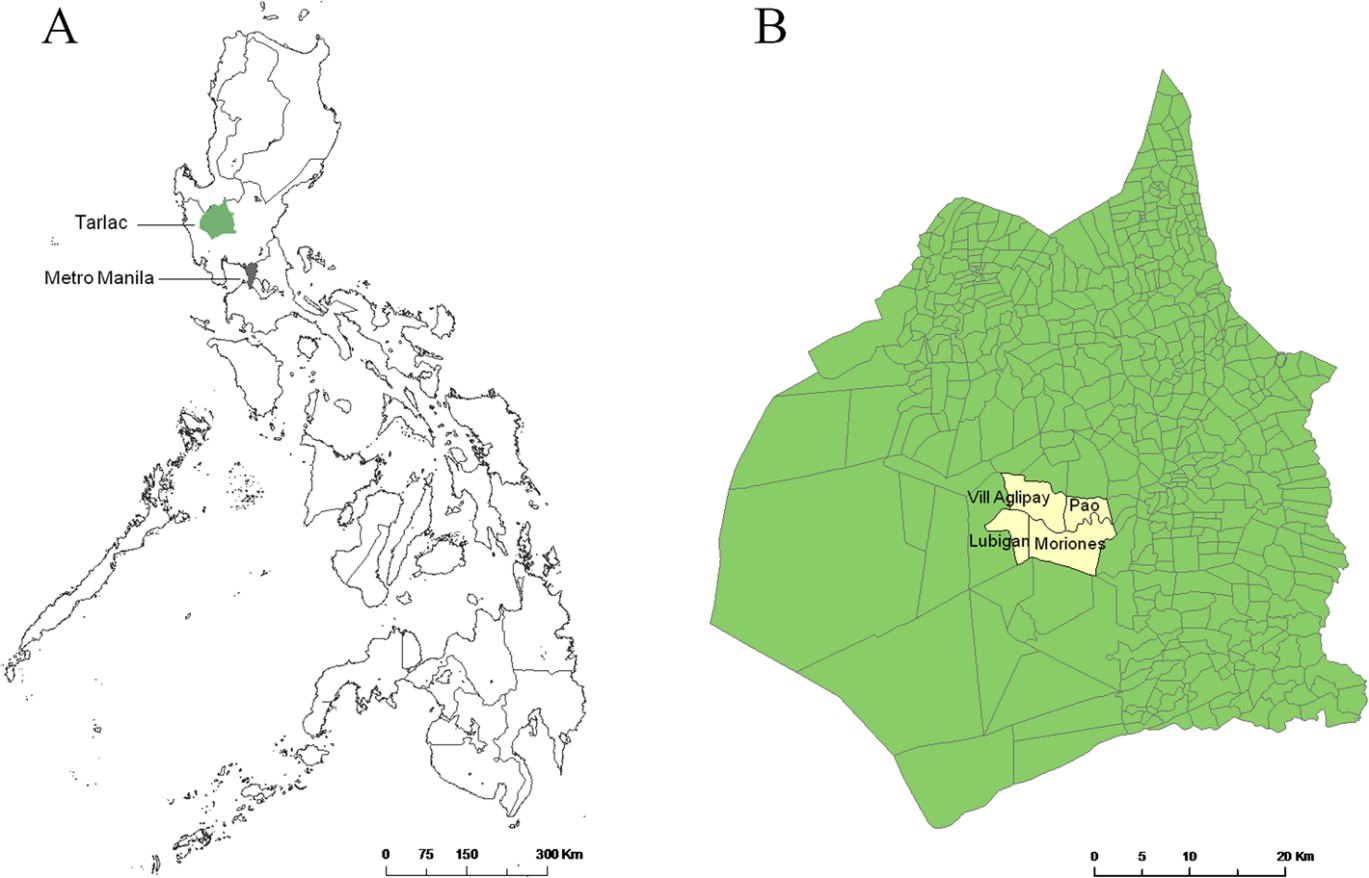
**Maps of study sites in the Philippines. A**. Tarlac Province (in green) is located north of the Philippines. **B**. Barangays of Pao, Villa Aglipay, Moriones, and Lubigan are located in the center of Tarlac Province.

The increasing documentation of zoonotic HEV in Asian countries such as China, Japan, Korea, Indonesia, Cambodia, Thailand, and Laos [15-17] suggests a significant health risk for the people. No epidemiological data are available regarding HEV infection among pigs or humans in the Philippines. However, recently, Li et al. reported that genotype 3 of HEV was found in the river water in Manila [18]. HEV infection in commercial pig farms were previously reported; however, there are very few reports on HEV infections in family-scale farms (backyard pig farms), where local people could be more frequently exposed to pigs or pig feces because of the open breeding system and poor sanitation of backyard pigs. The seroprevalence of HEV in family-scale pig farms was higher than that in large-scale pig farms as reported from Thailand [19] and China [20]. In rural areas of the Philippines, backyard pig farms are still quite common, and backyard pigs are an important source of income for pig owners. As a part of the project conducted in the Philippines to assess the prevalence of zoonotic pathogens, including Japanese encephalitis virus and Reston Ebola virus [21], we investigated the molecular characteristics and seroprevalence of HEV among household-raised pigs in four barangays (Villa Aglipay, Moriones, Pao, and Lubigan) in San Jose, Tarlac Province, the Philippines. Notably, San Jose is a third-class municipality and comprises mainly of rural areas in the Tarlac Province (Figure 1), where the density of household-raised pigs is quite high.

## Results

### Detection of anti-HEV IgG and IgM in pig sera and HEV RNA in stool swabs

Serum and rectal swab samples were collected from a total of 299 pigs aged 2–24 months (median age, 4 months) from 155 households in four barangays. The median numbers of pigs raised and numbers of samples per household were 2 [interquartile range 1–5] and 1[interquartile range 1–2], respectively. A majority of pigs were healthy, raised in simple piggeries in backyards, fed with commercial feeding or kitchen residues, and living with other domestic animals such as chickens and ducks. Anti-HEV IgG was found in 150 serum samples [50.3%, 95% confidence interval (CI) 44.5–56.2%] from 93 households (60.0%), with the similar average prevalence of 43.4–55.1% among four barangays (Table 1). Anti-HEV IgM was detected in 68 serum samples (22.9%, 95% CI 18.2–28.1%) from 52 households (33.5%). On the other hand, a total of 22 rectal swabs (7.4%, 95% CI 4.7–10.9%) from 16 households (10.3%) were positive for HEV RNA (Table 1). The average prevalence (56.2%, 95% CI 50.4–61.9%) of any of the three markers (anti-HEV IgG, IgM, and RNA) in the pig population was observed at similar range between 47.0% and 62.5% among the four barangays. Overall, 66.5% households (103/155, 95% CI 58.4–73.8%) had pigs positive for either anti-HEV IgG, IgM, or viral RNA. Among the 22 RNA positive samples, six samples were positive for both anti-HEV IgM and IgG and 10 samples were only positive for anti-HEV IgG. The remaining six samples were negative for both anti-HEV IgM and IgG.

**Table 1:** **The detection of anti-HEV IgG, IgM, and HEV RNA in household-raised pigs in four barangays**

**Barangay**	**No. of swine**	**% RNA (95% CI)**	**% IgG (95% CI )**	**% IgM (95% CI )**	**% one of three markers (95% CI)**	**No. of household**	**% household positive for one of three markers (95% CI)**
**Pao**	83	3.6 (0.8–10.2)	43.4(32.5–54.7)	22.0 (13.6–32.5)	47.0 (35.9–58.3)	49	53.1 (38.3–67.5)
**Villa Aglipay**	70	15.7 (8.1–26.4)	49.3 (37.0–61.6)	24.6 (15.1–36.5)	57.1 (44.7–68.9)	24	70.8 (48.9–87.4)
**Moriones**	98	7.1 (2.9–14.2)	55.1 (44.7–65.2)	21.4 (13.8–30.9)	60.2 (49.8–70.0)	52	73.1 (59.0–84.4)
**Lubigan**	48	2.1 (0.5–11.1)	54.2 (39.2–68.6)	25.0 (13.6–39.6)	62.5 (47.4–76.0)	30	73.3 (54.1–87.7)
**Total**	299	7.4 (4.7–10.9)	50.3 (44.5–56.2)	22.9 (18.2–28.1)	56.2 (50.4–61.9)	155	66.5 (58.4–73.8)

### The presence of anti-HEV IgG, IgM, and HEV RNA in different age groups

The prevalence of anti-HEV IgG (37.6%, 95% CI 30.3–45.2%) was the lowest in growing pigs (P < 0.0001) and then increased in finishing pigs (64.1%, 95% CI 53.6–73.9%) and reached a peak of 78.8% (95% CI 61.1–91.0%) in breeding pigs (Table 2). Also, the prevalence of anti-HEV IgM was the lowest in growing pigs (16.9%, 95% CI 11.6–23.3%), comparing to that in finishing pigs (27.2%, 95% CI 18.4–37.4%) and breeding pigs (42.4%, 95% CI 25.5–60.8%) (p = 0.05 and p = 0.0008, respectively). Growing pigs had the highest prevalence of viral RNA (9.2%, 95% CI 5.4–14.6%), followed by finishing pigs (5.4%, 95% CI 1.8–12.1%), and breeding pigs (3.0%, 95% CI 0.1–15.8%), although it was not statistically significant (p = 0.26 and p = 0.23, respectively).

**Table 2:** **The presence of anti-HEV IgG, IgM, and HEV RNA in pigs of different age groups**

**Age group of pigs**	**No. of pig**	**% RNA (95% CI)**	**% IgG (95% CI)**	**% IgM (95% CI)**
**Growing pigs (2–4 months)**	173	9.2 (5.4–14.6)	37.6 (30.3–45.2)	16.9 (11.6–23.3)
**Finishing pigs (5–7 months)**	93	5.4 (1.8–12.1)	64.1 (53.5–73.9)	27.2 (18.4–37.4)
**Breeding pigs (8–24 months)**	33	3.0 (0.1–15.8)	78.8 (61.1–91.0)	42.4 (25.5–60.8)
**Total**	299	7.4 (4.7–10.9)	50.3 (44.5–56.2)	22.9 (18.2–28.1)

### G**enetic analysis of HEV strains from stool swabs**

**Figure 2:**
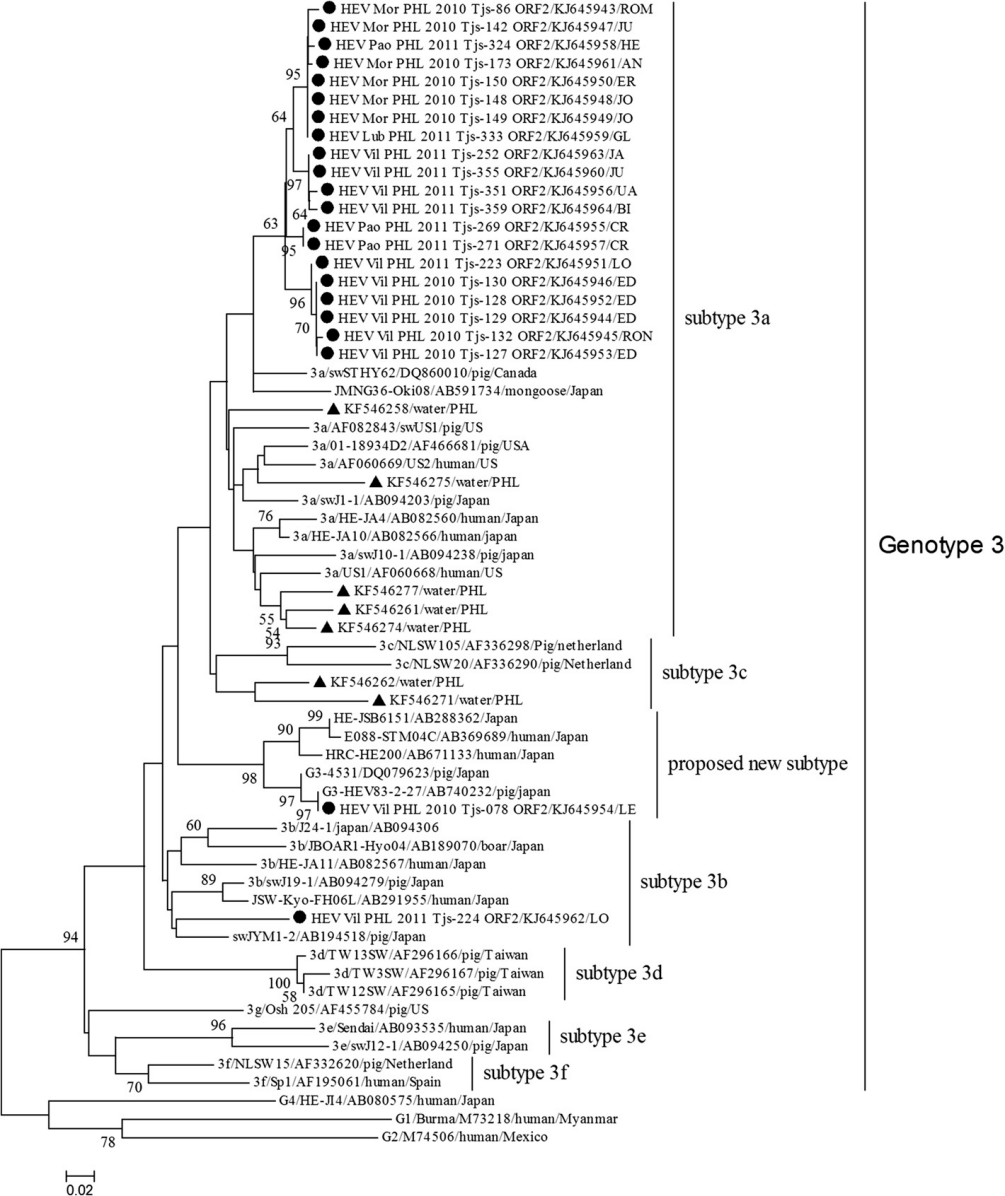
**Phylogenetic analysis of HEV strains from pigs in San Jose, Tarlac Province, the Philippines.** The phylogenetic tree was constructed using the neighbor-joining method (Kimura 2-parameter model) based on 301 nucleotides of ORF2 of HEV. Strains from this study were labeled with ● and tagged with household name (in capital letters) from where they originated. HEV genotype 3 strains from rivers in Manila, the Philippines, which were labeled with ▲, with GenBank accession numbers of KF546258, 546261, 546262, 546271, 546274, and 546277, were also included. Other reference strains were representatives of genotypes 1, 2, and 4 and subtypes 3a–3 g of genotype 3 in other countries. Reference strains were indicated as genotypes or subtypes, name, country, and GenBank accession number. The numbers on branches were bootstrap values (1,000 replicates; values less than 50% were not shown).

Phylogenetic analysis of 301 nucleotides corresponding to nucleotide positions 5994–6294 of M73218 in ORF2 revealed that 22 HEV strains in the Philippines belonged to genotype 3 (Figure 2). Pairwise comparison of 22 strains over 301 nucleotides revealed a 0–27.6% nucleotide difference. Compared with representative strains from river water in Manila, the strains in this study showed 10.0–24.0% nucleotide difference. With the exception of two strains (HEV_Vil_PHL_2011_Tjs-224_ORF2 and HEV_Vil_PHL_2010_Tjs-078_ORF2), the other 20 strains fell into a unique cluster within subtype 3a with a genetic distance of 0–4.9%. BLAST analysis revealed that these 20 strains shared less than 94% nucleotide similarities with any other sequence in GenBank. HEV_Vil_PHL_2011_Tjs-224_ORF2 shared the highest similarity (91%) with unclassified strains (JSW-Kyo-FH06L, AB291955) and subtype 3b strains (swJA11, AB082567) from Japan [22] and was also clustered with genotype 3b strains in the phylogenetic tree. The remaining strain HEV_Vil_PHL_2010_Tjs-078_ORF2 displayed 15.9–27.6% pairwise distance with other strains in this study. In the phylogenetic tree, it was clustered with unclassified reference strains from pigs (G3-HEV83-2-27 and G3-4531) and humans (HRC-HE200, HEJSB6151, and E088-STM04C) in Japan and shared 94–100% similarity with them. This cluster was genetically distant from other subtypes and may represent a novel subtype. Strains detected from the same household were closely clustered except two strains (HEV_Vil_PHL_2011_Tjs-223_ORF2 and HEV_Vil_PHL_2011_Tjs-224_ORF2). HEV_Vil_PHL_2011_Tjs-223_ORF2 and HEV_Vil_PHL_2011_Tjs-224_ORF2, which were collected in the same batch of pigs raised in the same household, genetically differed from each other by 15.4% and were grouped into subtype 3a and 3b in the phylogenetic tree, respectively.

## Discussion

We used the recombinant antigen (112–660 amino acids of ORF2) of one of the prototype strains of genotype 1 (GenBank accession number D10330), which proved to be effective in detecting HEV antibodies in both human and pig serum [23,24]. Notably, numerous commercial or in-house enzyme immunoassays, which were developed to detect antibodies in human sera, were also adapted to detect anti-HEV in pigs since only one serotype has been described [3,6,25]. We found that the anti-HEV IgG prevalence (50.3%) in pigs from rural communities of the Philippines was much higher than that in a similar study conducted in smallholder-raised pigs in rural villages of Laos (15.3%) [26] and comparable to that in large-scale surveys of pigs of all age groups from commercial pig farms in Japan (56%) [15], Germany (46.9%) [27] and Italy (50.2%) [28]. It is worthwhile to mention that in several other studies, discrepant results have been reported when comparing ELISAs using different antigens of HEV with human or porcine origin for detecting anti-HEV in pigs [29,30]. Compared with the prevalence of anti-HEV IgG, there are limited data on the seroprevalence of anti-HEV IgM in domestic pigs. The seroprevalence of IgM in this study (22.9%) was much higher than that in large-scale surveys in Japan (3%) [15] and similar to that in Spain (28.2%) [31]. Furthermore, 66.5% of households had at least one pig positive for anti-HEV IgG, IgM, or HEV RNA. In the present study, the piggeries of pigs were simple, in poor sanitary conditions and located at the household or near to the household. Such a high level of HEV infection in household-raised pigs, frequent exposure to pigs or pig waste, and poor sanitation in rural areas in the Philippines indicate potential risks of HEV transmission from pigs to local residents. Besides, the local people commonly consume the cooked pig livers, pork and sausages made by local manufactures. Therefore, workers in slaughterhouses and pork handlers in the local area are at potential risk of getting HEV infection. However, there are no available data on viral hepatitis incidence due to HEV in the Philippines. The human health impact of HEV should be properly defined to establish appropriate interventions.

Our data revealed that the seroprevalence of anti-IgG increased with age from 2–4-month-old pigs to 8–24-month-old pigs. A higher seroprevalence of IgG in adult pigs than in young pigs has also been documented in other studies [25,32]. However, according to antibody dynamics studies, there may be two seroprevalence peaks of anti-HEV IgG at less than one month old pigs due to maternal antibody and adult pigs in commercial pig herds [6,33-35]. In the present study, we did not observe the first peak of anti-IgG because the pigs in the present study were ≥2 months old and the maternal anti-IgG could persist up to 8–9 weeks of age in young pigs depending on the titers in breeding pigs [6,34,35]. It has been reported that seroconversion of IgM occurs in pigs aged 2–3 months and its duration varies from 4 to 7 weeks in commercial herds [6,34,35]. However, in some commercial herds, the peak prevalence of IgM was reported in pigs aged 25 weeks, which could be slaughtered, and IgM were also frequently detected in sows (up to 40%) [33]. We observed that the prevalence of IgM increased from 2–4-month-old pigs (16.9%) and reached a peak in 8–24-month-old pigs (42.4%); however, no infectious RNA was detected in rectal swabs of these breeding pigs except one. All these pigs were raised under poor sanitary condition, and breeding pigs usually lived with young pigs in rural communities. The high seroprevalence of IgM in breeding pigs was probably caused by the secondary immune response to frequent HEV exposure as reported among the vaccinated population exposed to measles virus [36]. This quick secondary immune response could prevent viral proliferation in the early phase; therefore, no RNA was detected. In the current study, we have provided important information about HEV infection status of pigs aged above 6 months while a majority of prevalence studies have been performed among pig aged less than 6 months. Six pigs with age ranging from 2–4 months were found negative for both IgG and IgM antibodies in sera but positive for HEV RNA in feces probably because of a recent infection [37]. On the other hand, in 10 pigs with age ranging from 2 to 8 months, RNA was detected in feces and IgG was found positive, but IgM was found negative. Detected IgG antibody might be due to persistent maternal antibody among 2–3 months old pigs (n = 5) and existed antibody from past infection in other five pigs aged 4–8 months. IgM negative results were probably because of a recent recurrent viral infection which could result in a low IgM immune response to HEV and a false negative result of IgM serological test. Moreover, it is possible that positive HEV in stools might reflect transient exposure to the virus through ingestion of contaminated food or water.

The average RNA-positive rate in rectal swab samples in this study (7.4%) was similar to that in Japan (5%) [15], Laos (11.6%) [16] and Thailand (2.9–7.75%) [19,38]. In this study, the viral RNA-positive rate was higher in 2–4-month-old pigs than in adult pigs, however the difference was not found statistically significant. Our finding is in line with other reports which stated that the highest incidence of HEV infections occurs in young pigs (2–4 months old) [15,39]. In Southeast Asia, both HEV genotypes 3 and 4 are circulating in human and swine; however, the geographical distribution of genotypes of zoonotic HEV in pigs varies. In Cambodia [40] and Thailand [19,38], only HEV genotype 3 has been reported in local pigs, while only genotype 4 of HEV has been reported in pigs from Laos [16]. In China [41], Japan [42], Korea [43], Indonesia [17], and Taiwan [44], both genotypes 3 and 4 were circulating in domestic pigs or wild boars. In the present study, we only detected HEV genotype 3 strains and a majority of them (20/22) were classified into the existing subtype 3a. Subtype 3a was also the most frequently detected subtype in Japan, Korea, and North America [4,45,46] as well as in river water in Manila in 2012 [18]. Subtype 3a strains in San Jose shared less than 94% nucleotide similarity with strains in GenBank (including strains from river water in Manila), which suggests that area-specific strains of HEV were circulating in the Philippines. HEV_Vil_PHL_2010_Tjs-078_ORF2 shares 100% sequence identity with the strain (G3-HEV83-2-27, AB740232) from a domestic pig in Japan in 2003; thus, HEV_Vil_PHL_2010_Tjs-078_ORF2 may have the same origin as the strain detected in a domestic pig in Japan. However, the exact transmission route of this virus remains unknown. HEV_Vil_PHL_2010_Tjs-078_ORF2 together with G3-HEV83-2-27 and some unclassified strains from Japan formed a distinct cluster from other subtypes. The full sequence of G3-HEV83-2-27 is available in GenBank (accession number AB740232), and it can also form a distinct cluster in a phylogenetic tree based on the full genome sequence (see Additional file 1: Figure S1). Therefore, this cluster, including HEV_Vil_PHL_2010_Tjs-078_ORF2, could represent a new subtype.

## Conclusion

The present study is the first report on the seroprevalence and molecular characterization of HEV in pigs in the Philippines. We found a high proportion of IgG and IgM, and three different subtypes of HEV among household-raised pigs suggesting that the risk of HEV transmission to humans in this geographical area was substantial. Hepatitis E is not included as a notifiable disease in the Philippines, and laboratory testing for acute hepatitis is not routinely performed in the country. Since only pig population from a small geographic area were investigated in the present study, further studies are required to define the genotype distribution in other areas, genetic relationship between HEV strains from swine and human and human health impact of HEV in the Philippines.

## Methods

### Samples

Swine blood and rectal swab samples from previous cross-sectional survey for validation of an ELISA assay [21] were tested for prevalence of HEV at the research institute for tropical medicine in Manila, Philippines. The sample collection was divided in six phases between July 2010 and June 2011. Households known to have pigs in their backyard farms were visited and owners were asked for the participation into the study. Informed consent was obtained from the pig owners. The pigs were stratified by age in months and selected to have all age groups available in each household. Piglets less than two months old were excluded from the sampling. The sampling was not systematic or random. Up to 50 samples were collected per sampling phase. This study was approved by ethics committee of Research Institute for Tropical Medicine and Institutional Animal Care and Use Committee at the Animal Welfare Division of Bureau of Animal Industry, the Department of Agriculture.

### Assessment of HEV infection by serology

To detect anti-HEV IgM and IgG, ELISA was performed. Virus-like particles (VLPs) were expressed by a recombinant HEV Burma strain (genotype 1) ORF2 (112–660 amino acids of D10330) using baculovirus in Tn5 cells, as described previously [47]. Flat-bottom 96-well polystyrene microplates (Becton, Dickinson and Company, NJ, USA) were coated with purified VLPs (1 μg/ml, 100 μl/well) and incubated at 4°C overnight. Unbound VLPs were washed out with 300 μl of 10 mM phosphate-buffered saline containing 0.05% Tween 20 (PBS-T). The wells were blocked for 1 h with 200 μl of 5% skim milk (Becton, Dickinson and Company, NJ, USA) in PBS-T at 37°C. After the plates were washed three times with PBS-T, swine serum samples (100 μl/well) were added in duplicate at a dilution of 1:200 in PBS-T containing 5% skim milk. The plates were then incubated for 1 h at 37°C. The plates were washed three times as described above and were administered 100 μl of horseradish peroxidase-conjugated goat anti-pig IgG (Bethyl, Laboratories, Inc., TX, USA) (1:10,000 dilution) or IgM (Kirkegaard & Perry Laboratories, Inc., MD, USA) (1:2,500 dilution) in PBS-T containing 1% skim milk. The plates were incubated for 1 h at 37°C and washed three times with PBS-T. Subsequently, 100 μl of o-phenylenediamine dihydrochloride (Sigma-Aldrich, Co., MO, USA) was added to each well. The plates were incubated in a dark room for 30 min at room temperature, following which 100 μl of 4 N H_2_SO_4_ was added to each well. The optical density was measured at 492 nm. Four standard deviation values above the mean OD value of negative controls (n = 4) were applied as the cut-off value for each plate.

### Detection and genotyping of HEV infection

Rectal swabs were soaked in a viral transport medium containing Hank’s balanced salt solution supplemented with gelatin, streptomycin, penicillin-G, and amphotericin B. RNA was extracted from 140 μl of sample using the QIAamp MinElute Virus Spin Kit (Qiagen, Hilden, Germany). Reverse transcription was performed using SuperScript III reverse transcriptase with a random hexamer (Life technologies, Carlsbad, CA, USA). A previously described nested polymerase chain reaction (PCR) [8] was performed to amplify part of ORF2, which corresponds to the nucleotide positions 5939–6316 of the genotype 1 HEV genome (GenBank accession number M73218). The PCR products were purified using the QIAquick® PCR Purification Kit (Qiagen), sequenced using BigDye Terminator version 1.1 (Life technologies), and analyzed using Applied Biosystems 3700 Genetic Analyzer (Life technologies). HEV genotypes were determined by phylogenetic analysis using MEGA (version 5) [48]. The pairwise distance was calculated by the neighbor-joining method, and the phylogenetic tree was constructed by the Kimura 2-parameter model, neighbor-joining method by MEGA 5. Strains in this study were named as HEV_ Barangay code_PHL _year_ID number of strain_ORF2 and were deposited in the GenBank database under the accession number of KJ645943–KJ645964. The GenBank accession numbers of reference strains are given as follows: DQ860010, AB082566, AB194492, AF060669, AB740232, DQ079632, AB671133, AB369689, AB288362, AB291955, AB094279, AB082567, AF296167, AF296165, AF336298, AF336290, AF336293, AB093535, AF332620, AF455784, AB080575, AF296166, AB094250, AF195061, AB591734, AB291955, AB194518, M73218, and M74506.

### Availability of supporting data

The data set supporting the results of this article is included within the article and its additional file.

### Statistical Analysis

Pigs in this study were classified into three age groups according to local pig production stage: growing pigs (2–4 months), finishing pig (5–7 months), and breeding pigs (8–24 months). The prevalence of anti-HEV IgG, IgM and HEV RNA between different age groups were compared by using Chi-square test in Stata software, version 12 (StataCorp, College Station, Texas), p ≤ 0.05 was considered statistically significant.

## Additional file

Additional file 1: Figure S1. Phylogenetic analysis of HEV based on complete genome. The phylogenetic tree was constructed by the neighbor-joining method with the Kimura 2-parameter model. The strain G3-HEV83-2-7 (sharing 100% similarity in 301 nucleotides with HEV_Vil_PHL_2010_Tjs-078_ORF2) with ♦ label could represent a new subtype under genotype 3 based on full genome sequence analysis.

## Abbreviations

HEV: Hepatitis E virus
ORF: Open reading frame
nt: Nucleotide
ELISA: Enzyme-linked immunosorbent assay
PCR: Polymerase chain reaction
VLP: Virus-like particle
Ig: Immunoglobulin
PBS-T: Phosphate buffered saline containing 0.05% Tween 20
